# Stepping into 2025: highlights of the February issue

**DOI:** 10.1192/bji.2024.41

**Published:** 2025-02

**Authors:** Hussien Elkholy

**Affiliations:** Clinical Assistant Professor in Psychiatry, Department of Clinical Neuroscience, Brighton and Sussex Medical School, Brighton, UK. Email: h.elkholy2@bsms.ac.uk

Approaching the end of 2024, I am about to send this editorial to the publisher. This made me reflect on my short time with *BJPsych International* and how I have witnessed its evolution and the recently awarded impact factor of 2.1. As the Deputy Editor, I am proud to be part of the family of a continuously growing and evolving journal.

In this issue, *BJPsych International* continues to be a global platform acknowledging contributions from everywhere, especially low- and middle-income countries. An article by Smekhov discusses current mental health services in Uzbekistan and their recent reforms.^1^ The article focuses on six key domains: service delivery, the healthcare workforce, health information systems, access to essential medicines, financing and governance.

The issue also has two interesting articles on child psychiatry. In the first of these Sultan discusses barriers children with autism face in accessing equitable and sustainable healthcare, including geographical disparities, cultural stigma, communication barriers and inadequate training of healthcare providers. The author encourages investing in telehealth, supporting financial assistance programmes and adopting culturally competent, tiered care systems.^2^ On a similar note, Reba et al discuss the concept of metacommunity and the role of psychiatrists in strengthening and influencing mental health policies.^3^

The second child psychiatry article highlights the difficulty of studying uncommon mental health problems and sheds the light on the development of the Royal College of Psychiatrists’ Child and Adolescent Psychiatry Surveillance System (CAPSS).^4^

Psychiatry is a rapidly evolving specialty, and digital psychiatry is a field that is receiving more attention in this technological era. Unfortunately, this also opens the doors to digital applications that may not have any evidence base. An interesting article by Sethi et al^5^ examines the current regulatory landscape for mental health apps in India, compares international approaches and proposes a regulatory framework.

Hikikomori is a severe form of social isolation that was previously believed to be specific to Japan. Over the past few years increased attention has been paid to it, as many cases have been reported worldwide. Nagai et al take us on journey to understand the evolution of the term and its cultural significance as it becomes a global phenomenon.^6^

Finally, training is a main pillar in developing and improving services. Owing to lack of aftercare and community mental health services, drug addiction relapses in Nepal are as high as 75% during the 6 months following treatment. Jha et al share the experience of ‘training the trainers’ in a mindfulness-based relapse prevention approach.^7^

## Funding

This research received no specific grant from any funding agency, commercial or not-for-profit sectors.

## Declaration of interest

H.E. is a member of the *BJPsych International* editorial board and did not take part in the review or decision-making process of this paper.
